# Impaired Caveolae Function and Upregulation of Alternative Endocytic Pathways Induced by Experimental Modulation of Intersectin-1s Expression in Mouse Lung Endothelium

**DOI:** 10.1155/2012/672705

**Published:** 2012-02-26

**Authors:** Dan N. Predescu, Radu Neamu, Cristina Bardita, Minhua Wang, Sanda A. Predescu

**Affiliations:** ^1^Department of Pharmacology, Rush University, 1735 W Harrison, Chicago, IL 60612, USA; ^2^Division of Pulmonary, Allergy and Critical Care Medicine, Emory University School of Medicine, Atlanta, GA 30322, USA

## Abstract

Intersectin-1s (ITSN-1s), a protein containing five SH3 (A-E) domains, regulates via the SH3A the function of dynamin-2 (dyn2) at the endocytic site. ITSN-1s expression was modulated in mouse lung endothelium by liposome delivery of either a plasmid cDNA encoding myc-SH3A or a specific siRNA targeting ITSN-1 gene. The lung vasculature of SH3A-transduced and ITSN-1s- deficient mice was perfused with gold albumin (Au-BSA) to analyze by electron microscopy the morphological intermediates and pathways involved in transendothelial transport or with dinitrophenylated (DNP)-BSA to quantify by ELISA its transport. Acute modulation of ITSN-1s expression decreased the number of caveolae, impaired their transport, and opened the interendothelial junctions, while upregulating compensatory nonconventional endocytic/transcytotic structures. Chronic inhibition of ITSN-1s further increased the occurrence of nonconventional intermediates and partially restored the junctional integrity. These findings indicate that ITSN-1s expression is required for caveolae function and efficient transendothelial transport. Moreover, our results demonstrate that ECs are highly adapted to perform their transport function while maintaining lung homeostasis.

## 1. Introduction

ITSN-1s is a multimodular protein, evolutionary conserved and widely expressed [1, 2]; it consists of two NH_2_-terminal EH domains, a central coiled-coil domain, and five consecutive COOH-terminal SH3 domains, SH3A-E, [3, 4]. Similarly to Dyn, ITSN-1s localizes to endocytic clathrin-coated pits and caveolae at the plasma membrane and associates preferentially with the neck region of caveolae [5, 6]. The simultaneous presence of multiple SH3 and EH domains, best known for their function in endocytosis, as well as the subcellular localization of ITSN-1s, led to the early assumption that ITSN-1s may function as an adaptor/scaffold of the general endocytic machinery [3, 5]. Subsequent studies have shown that ITSN-1s is capable of binding essential endocytic proteins, Epsin1/2, Eps15 [7], both the neuronal and ubiquitously expressed Dyn isoforms, [3, 5, 6, 8], stonin 2 [9, 10], and the signaling proteins RaIBP-associated Eps15-homology domain protein [11], mSos [12, 13], and 5-phosphatase SHIP2 [14]. ITSN-1s binds several Dyn molecules simultaneously and clusters them at the endocytic sites, creating a high concentration of Dyn required for collar formation around the necks of endocytic vesicles [6, 8]. This is a crucial endocytic event since the GTPase activity of Dyn is allosterically dependent on Dyn protein concentration [15, 16] and GTP-dependent self-assembly of Dyn into rings around the necks of budding vesicles plays a key role in membrane fission [17, 18]. Protein overexpression, loss-of-function mutations, knockdown or knockout approaches have provided compelling evidence for the crucial role of ITSN-1s in scaffolding the general endocytic machinery, recruitment of Dyn, and regulation of its function at the endocytic sites [6, 8, 16, 19–21]. Evidence for a possible role of ITSN-1s in regulation of Dyn function during synaptic vesicles endocytosis has been reported in different species and experimental conditions [16, 21–23]. The ortholog of mammalian ITSN-1s in Caenorhabditis elegans has a negative regulatory effect on a Dyn-controlled pathway or on Dyn itself [22]. Drosophila's ITSN, Dyn-associated protein (Dap160), scaffolds endocytic machinery and promotes efficient endocytosis by maintaining a high concentration of Dyn and allowing allosteric activation of its GTPase activity at synapses [15, 16, 23]. Moreover, in lamprey giant reticulospinal synapse, acute perturbation of ITSN-Dyn interaction suggested that ITSN may control the amount of Dyn released from the synaptic vesicle cluster to the periactive zone and that ITSN may regulate fission of clathrin-coated intermediates [21]. ITSN-1s knockout mice established the requirement of ITSN-1s for efficient synaptic vesicle endocytosis and trafficking [20]. Moreover, the SH3A domain of ITSN-1s has the unique ability to regulate Dyn2 oligomerization and its GTPase activity at the neck region of caveolae [8]. Overexpression of full-length or truncated ITSN-1s in cultured ECs caused severe impairment of caveolae endocytic function. Electron microscopy analysis of these cells revealed a wide range of morphological changes such as caveolae with pleomorphic necks, many of them surrounded by Dyn collars, caveolae clustering, and impaired membrane fission [6, 8]. In particular, expression of the SH3 region, assumed to function in a dominant negative manner by sequestering Dyn away from the caveolae endocytic sites caused significant decrease in caveolae number [6]. Recent studies revealed the association of ITSN-1 with arfaptin2, Src homology 3 domain Growth factor receptor-bound 2-like (endophilin) Interacting Protein 1 (SGIP1), and Fer/Cip4 homology domain only proteins 1 and 2 (FCHO) with membrane-deforming/bending activity essential for initiating clathrin-coated pits maturation and coated vesicle formation [11, 24, 25].

In the present study, we addressed the *in vivo* role of ITSN-1s in the regulation of caveolae function in transendothelial transport by experimental modulation of ITSN-1s expression in mouse lung endothelium. Our findings provide evidence that ITSN-1s is required for efficient caveolae functioning during transendothelial transport. Moreover, modulation of ITSN-1s expression and the subsequent impairment of caveolae internalization and trafficking upregulated alternative endocytic/transcytotic pathways to compensate for deficient caveolae function.

## 2. Materials and Methods

### 2.1. Materials

Cholesterol and dimethyl dioctadecyl ammonium bromide (DDAB) and bovine serum albumin (BSA) were from Sigma (St. Louis, MO). MicroBCA protein assay kit and enhanced chemiluminescent substrate (ECL) were from Pierce (Rockfort, IL). Relevant antibodies (Abs) were purchased as follows: rabbit anti-DNP polyclonal Ab from Invitrogen (Carlsbad, CA), ITSN-1 mAb from BD Transduction Laboratories (Lexington, KY), *α*-actin monoclonal Ab from Sigma, and myc mAb from Cell Signaling (Danvers, MA). SureBlue Reserve TMB Microwell Peroxidase Substrate and horseradish-peroxidase- (HRP-) conjugated reporters were from KPL (Gaithersburg MA). HyBlot CL films were from Denville Scientific Inc., (Metuchen, NJ). All electron microscopy reagents were from Electron Microscopy Science (Forth Washington, PA). The tracers, DNP-BSA, and 8 nm-BSA complexes were prepared in our laboratory as previously described [26, 27]. Full-length human ITSN-1 cDNA (Gift from Suzana de la Luna, Center for Genomic Regulation, UPF, and Centro de Investigacion Biomedica en Red de Enfermedades Raras (CIBERER-ISCIII), Barcelona, Spain), was used as a template to generate C-terminal myc-his-SH3A (residues 740–806) as in [8]. SMARTpool reagents and ITSN-1 siRNA duplex [GGACAUAGUUGUACUGAAAUU (sense sequence) and 5′-UUUCAGUACAACUAUGU CCUU (antisense sequence)] for knocking down ITSN-1s expression were from Dharmacon (Lafayette, CO).

### 2.2. Animals

 CD1 male mice, 6–8 weeks old, 20–25 g weight, from Jackson Laboratory (Bar Harbor, ME), kept under standardized housing and feeding conditions, were used. The experiments were done under anesthesia, using ketamine (60 mg/kg), acepromazine + xylasine (2.5 mg/kg) + (2.5 mg/kg), and xylazine (2.5 mg/kg) in 0.1-0.2 mL PBS. All experiments were approved and performed in accordance with the guidelines of Rush University Institutional Animal Care and Use Committee.

### 2.3. Protein Extraction

 Mouse lung tissue was homogenized in a buffer containing 150 mM NaCl, 50 mM Tris, pH 8.0 and protease inhibitors; total lysates were prepared by adding NP-40, to a final concentration of 1.0%, for 2 h, at 4°C. The resulted lysates were clarified by centrifugation in a Beckman tabletop ultracentrifuge with a TLA-55 rotor, for 45 min, at 4°C and 45,000 rpm. Protein concentration was determined by the microBCA method with a BSA standard.

### 2.4. Western Blot and Densitometry

 Equal amounts of total protein (70 *μ*g/lane) were subjected to 4–20% SDS PAGE, transferred to nitrocellulose membranes, and then subjected to Western blot analyses as in [28], using Abs against ITSN-1s, myc tag, as well as actin as loading control. The bands of immunoreactivity were visualized using an appropriate HRP-conjugated reporter Ab and ECL detection. HyBlot CL films were subjected to densitometric analysis using NIH ImageJ1.37v software.

### 2.5. Cationic-Liposome-Mediated Delivery of Myc-SH3A and ITSN-1s siRNA to Mouse Lungs

 Cationic liposomes were prepared using DDAB and cholesterol by rotary evaporation of lipid solutions in chloroform, at 42°C, under vacuum as in [29, 30]. The lipid film was rehydrated for 1 h at RT in 5% dextrose followed by sonication and sterile filtration through 0.47 *μ*m and 0.22 *μ*m membranes. Then, the suspension was extruded through a 50 nm polycarbonate filter mounted on a miniextruder device (Avestin, Inc., ON, Canada), to assure unilamellar vesicles formation. DNA-liposomes complexes were prepared using ratio 8 nmoles liposomes: 1 *μ*g myc-SH3A DNA. For silencing ITSN-1 gene expression in mouse lungs we used Dharmacon *SMART*pool reagents, as in [19]. RNase free conditions and reagents were used throughout the silencing experiments. Preliminary experiments were performed to identify the siRNA sequence of the *SMART*pool, most efficient in silencing ITSN-1s gene. Briefly, the siRNA sequences for knocking down ITSN-1s protein expression were first delivered in cultured mouse fibroblasts using Dharmafect Transfection Reagent according to the manufacturer's instructions. The target sequence 5′-GAGAGAGCCAAGCCG GAAA-3′ was found to be the most efficient in downregulation of ITSN-1s expression in mouse fibroblasts and thus, used to generate the siRNA duplex for *in vivo* delivery. Also, the si*CONTROL *functional nontargeting siRNA sequence 5′-UAGCGACUAAACACAUCAA-3′ was used as a control for potential secondary effects caused by siRNA transfection of mouse fibroblasts.

For silencing ITSN-1s gene, siRNA/liposomes complexes we prepared using 100 *μ*g ITSN-1s/mouse, 1 : 3 (vol/vol) siRNA:liposomes ratio. Mice were injected intravenously (tail vein) with cationic liposomes complexes, to achieve either transduction of the specific myc-SH3A construct or knockdown of ITSN-1s in lung microvessels. The lungs were collected at different time points after the first delivery of myc-SH3A plasmid or ITSN-1s siRNA/liposomes complexes to monitor myc-SH3A and ITSN-1s protein expression levels.

### 2.6. DNP-BSA and 8 nm Au-BSA Perfusion

 DNP-BSA (10 mg/mL) was perfused as in [26], to assess lung vascular permeability to albumin in control mice, mice expressing the SH3A, or ITSN-1s-deficient mice. The biochemical quantification of transendothelial transport was done as in [26]. Briefly, after tracer perfusion, the lungs were hand minced and homogenized in PBS, using a Brinkmann polytron (1 min, 4°C). The homogenates were centrifuged (45,000 rpm, 45 min) to obtain the final supernatants, expected to contain the lung interstitial fluid and thus, the DNP-derivatized BSA transported to the lung interstitia. The amounts of DNP-BSA were assessed by ELISA using HRP-conjugated anti-DNP Ab followed by a colorimetric assay with TMB microwell peroxidase substrate as in [26]. The plates were read in an ELISA reader at 650 nm. For quantitative assessment of DNP-BSA transendothelial transport, a standard curve using known concentration of DNP-BSA was generated. The data were expressed in ng DNP-BSA normalized per mg total protein and 10 min. Au-BSA complexes (A_520_ = 1.0) were perfused *in situ* for 10 min, the unbound tracer was flushed out by 3 min perfusion using oxygenated PBS as in [27].

### 2.7. RT-PCR

Mouse lung total RNA was extracted using RNeasy Mini Kit with on-column DNase digestion by RNase-free DNase set (Qiagen, Valencia, CA). The cDNA was synthesized by using the High-Capacity Reverse Transcription Kits (Applied Biosystems, ABI, Carlsbad, CA). The RT-PCR reactions were amplified for 35 cycles with the appropriate primer sets for the target genes, mouse ITSN-1s, ITSN-2s and ITSN-2L, and a house-keeping gene cyclophilin by using GoTag Green Master Mix (Promega, San Luis Obispo, CA). Two to 10 *μ*L of PCR products were subjected to 1.2% agarose gel electrophoresis and visualized using an Alpha Innotech AlphaImager II system. Primer pairs (Sigma, San Louis, MO) used in PCR: mouse ITSN-1s (forward: 5′-CAGGCTTGAAAAGTCCTCAAA-3′, reverse: 5′-GGTGATCATGCTGGAAG TCA-3′); mouse ITSN-2s (forward: 5′-CTGGGCACAATGCAGCCTACG-3′, reverse: 5′-CA AAAAGTTGCAGGCCGTCCA TG-3′), mouse ITSN-2L (forward: 5′-CCAGGACGTTCT GTGTCTCACTATG-3′, reverse: 5′-CAGTAGTAGTCGGCGGGTGGT-3′), and mouse Cyclophilin (forward: 5′-GGCAAATGCTGGACCAAACAC-3′, reverse: 5′-TTCCTGGAC CCAAA ACGCTC-3′).

### 2.8. Real-Time qPCR

 reactions were performed using Power SYBR Green PCR Master Mix (ABI) and analyzed in triplicate with a MyiQ Single Color Real-Time PCR Detection System (Bio-Rad, Hercules, CA). The relative expression level for each gene was calculated by a mathematical delta-delta Ct method developed by Applied Biosystems. The amounts of RNA were normalized to the house-keeping gene cyclophilin for all time points and reported relative to control mouse lungs (set at 1).

### 2.9. Electron Microscopy

 8 nm Au-BSA perfused mouse lungs were fixed *in situ* by 15 min with 4% (wt/vol) formaldehyde and 2.5% glutaraldehyde in 1% tannic acid in 0.1 PIPES buffer pH 7.2, and then excised and fixed for 1 h at RT, in the same fixative mixture. Fixed lungs were used to collect small tissue blocks that were processed for electron microscopy by standard procedures including 4% OsO_4_ alter fixation as in [26, 27]. Selected specimens were dehydrated in increasing concentrations of ethanol, followed by propylene oxide, and embedded in Epon 812. Thin sections cut from blocks were mounted on nickel grids, then stained with uranyl acetate and lead citrate, and finally examined and micrographed in a Joel 1220 transmission electron microscope.

### 2.10. Morphometric Analyses

 Morphometric analyses of normal and altered caveolar profiles as well as nonconventional endocytic/transcytotic structures were performed as described previously [6]. Briefly, 15–20 sections per grid and 3–5 grids per block, from 8 Epon812 blocks, chosen at random from control and ITSN-1s siRNA-treated mice, were used. The morphological appearance was recorded on 150 micrographs (×30,000 standard magnification) for each experimental condition. The measurement of the length plasma membrane on the electron micrograph used was performed as in [31]. The data were normalized per 100 *μ*m length plasma membrane and are averages ± SD of three independent experiments.

### 2.11. Statistical Analysis

 Data were compared using one way analysis of variance, Student's *t*-test with a Bonferoni correction for multiple comparisons, and a two-way analysis of variance where needed. Statistical significance was set at *P* < 0.05.

## 3. Results

### 3.1. Experimental Manipulation of ITSN-1s Expression in Mouse Lungs

**Figure 1:**
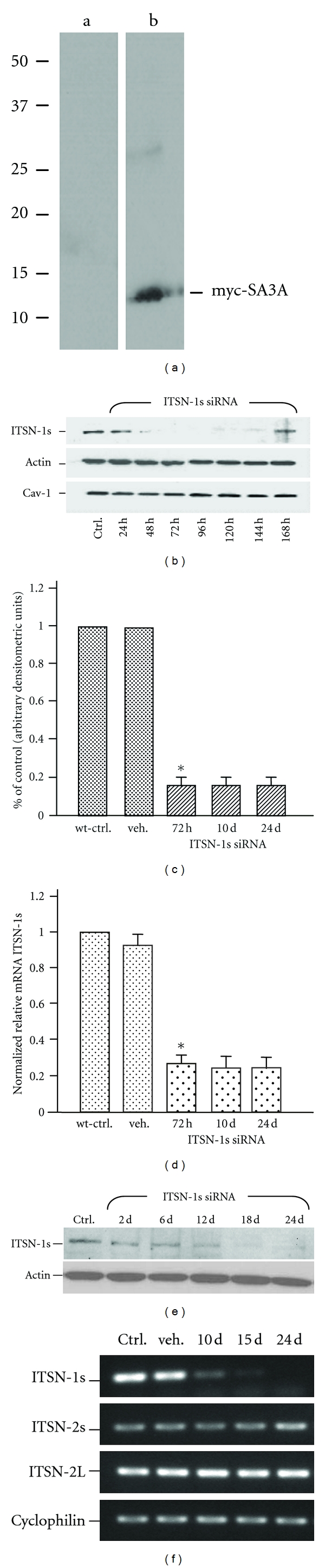
Experimental modulation of ITSN-1 expression in mouse lungs. (a) Myc-SH3A domain of ITSN-1s is efficiently expressed in mouse lungs, at 48 h alter delivery of the plasmid cDNA/liposome complexes. Mouse lung lysates (70 *μ*g total protein/lane) of control and myc-SH3A-transduced mice were subjected to 4–20% SDS PAGE, transferred to nitrocellulose membrane and immunoblotted with myc Ab. (b) siRNA-mediated knockdown of ITSN-1s in mouse lungs. Lung lysates (70 *μ*g total protein/lane) of controls and mice injected with liposomes/ITSN-1s siRNA complexes were resolved on 4–20% SDS PAGE and the electrophoretograms were transferred to nitrocellulose membrane followed by Western blot with Abs against ITSN-1, actin (as a loading control) and cav-1. Note the efficient knockdown of ITSN-1s protein for 96 h, starting at day 3 (3 d) alter siRNA delivery. (c) Densitometric analysis of representative HyBlot CL films demonstrates that treatment with ITSN-1s siRNA/liposome complexes downregulates efficiently ITSN-1s protein in mouse lungs. (d) Quantitative RT PCR of ITSN-1s mRNA in ITSN-1s siRNA treated mouse lungs. Values were normalized to the house-keeping gene cyclophilin and reported relative to control lungs (set at 1). (e) Chronic inhibition of ITSN-1s expression for 21 consecutive days by repeated delivery of ITSN-1s siRNA/liposomes complexes. Lung lysates of control and ITSN-1s-siRNA-treated mice were analyzed by Western blot with Abs against ITSN-1s and actin, as loading control, at several time points alter delivery, as indicated. (f) Conventional RT-PCR of ITSNs isoforms expressed in mouse lungs. RT PCR reactions were amplified as described under Materials and Methods using appropriate primer sets for the target genes, ITSN-1s, ITSN-2s and ITSN-2L, and a house-keeping gene cyclophilin. 5-*μ*L of PCR products were subjected to 1.2% agarose gel electrophoresis and visualized using an Alpha Innotech AlphaImager II system. All data shown are representative for 3 different experiments, (3 mice/experimental condition).

To evaluate *in vivo* the role of ITSN-1s in regulating caveolae function during transendothelial transport we modulated the expression of ITSN-1s in mouse lung endothelium by liposome delivery of a plasmid cDNA encoding the SH3A domain, or a specific siRNA duplex targeting ITSN-1 gene. Myc-SH3A DNA plasmid : liposomes complexes, prepared as described under Materials and Methods, were delivered intravenously to mouse lungs as in [29, 30]. The lungs were collected at different time points after the first delivery of myc-SH3A lipoplexes to monitor myc-SH3A protein expression. Western blot of mouse lung lysates using myc mAb indicated efficient SH3A expression, at 48 h alter DNA treatment (Figure 1(a)). For ITSN-1s knockdown, the silencing effect of ITSN-1s siRNA started at 48 h, it was obvious at 72 h alter siRNA administration and lasted for an additional 72 h, as evaluated by Western blot of mouse lung lysates with ITSN-1 mAb (Figure 1(b)). ITSN-1s protein expression recovered at 168 h alter siRNA delivery. Actin was used as a loading control. ITSN-1s knockdown did not affect the expression of caveolin-1 (Cav1), the structural protein of caveolae coat [32]. Densitometric analyses indicated a greater than 70% reduction of ITSN-1s protein levels at day 3, compared to wild-type untreated mice (Figure 1(c), asterisk). Intravenous injection of empty liposomes did not affect ITSN-1s protein levels (not shown). Efficient knockdown of ITSN-1s at 72 h following siRNA delivery was further confirmed by real-time quantitative (q) PCR (Figure 1(d)). ITSN-1s mRNA levels in ITSN-1s siRNA-treated mouse lung samples were more than 3-fold lower by reference to controls (Figure 1(d), asterisk). Delivery of empty liposomes (vehicle) did not affect the levels of mRNA in mouse lungs, compared to untreated mice. To get more insights into the effects of ITSN-1s deficiency on caveolae function we also generated a mouse model with chronic inhibition of ITSN-1s, by repeated delivery of ITSN-1s siRNA/liposome complexes, every 72 hr, for 24 consecutive days (Figure 1(e)). Western blot analyses applied on mouse lung lysates of ITSN-1s siRNA-treated indicated continuous knockdown of ITSN-1s for 21 days. For accurate quantitative data, we used again qPCR analysis. The results indicated that the real knockdown of the target ITSN-1 gene was about 3-fold by reference to cyclophilin, the gene used as standard (Figure 1(d), double asterisk). In addition, conventional RT PCR applied on lung samples prepared from controls and ITSN-1s-deficient mice (Figure 1(f)) demonstrated that the knockdown of ITSN-1s is specific and efficient. The expression of ITSN-1s mRNA was not detected at days 10, 15 and 24 alter ITSN-1s siRNA delivery, while ITSN-2s and ITSN-2L mRNAs were not detectably affected by comparison to untreated controls. These murine models generated by liposome delivery of the cDNA plasmid encoding the SH3A domain and the ITSN-1s siRNA were used for a further in-detail study of ITSN-1s endocytic function.

### 3.2. Acute Perturbation of ITSN-1s Expression Induces Pleomorphic Endocytic/Transcytotic Structures and Increases Mouse Lung Vascular Permeability

**Figure 2:**
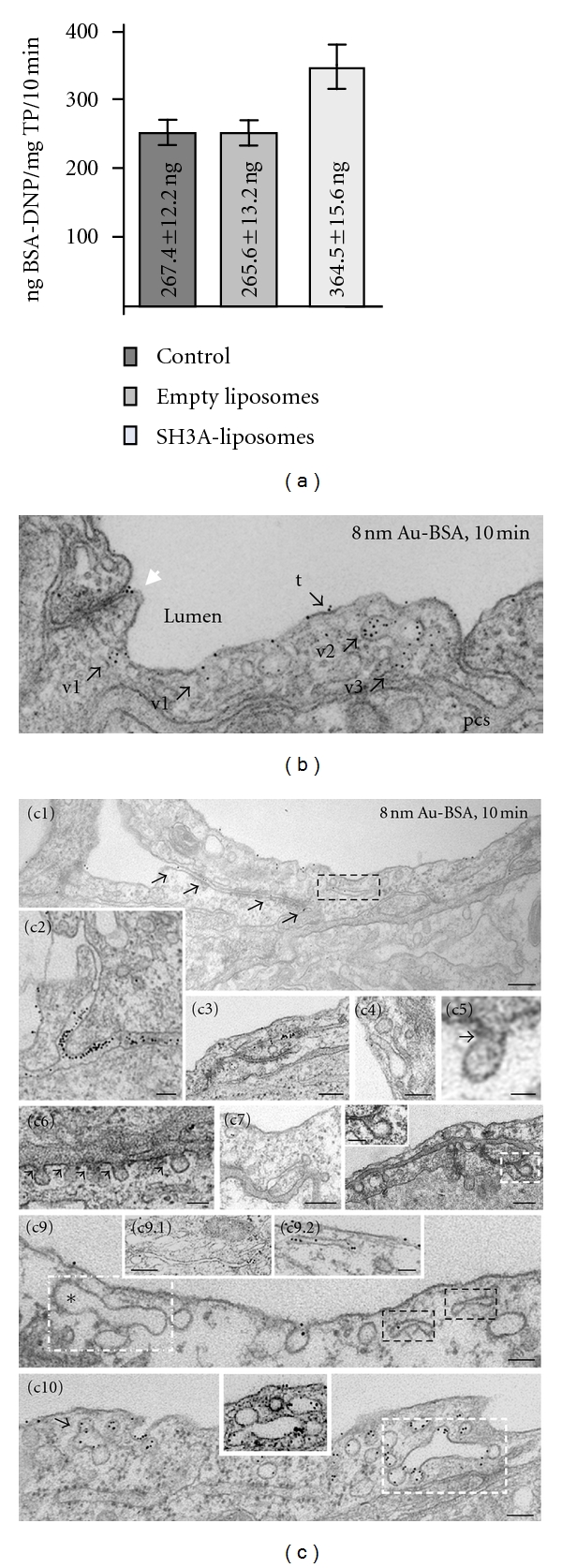
Increased endothelial permeability, impaired membrane fission, and increased occurrence of nonconventional endocytic/transcytotic intermediates in the mouse lungs expressing the SH3A domain of ITSN-1s. (a) ELISA applied on mouse lung lysates of control, empty liposomes, and myc-SH3A-transduced mice perfused with 10 mg/mL BSA-DNP, for 10 min. Results were expressed in ng BSA-DNP/mg total protein/10 min. Bars ± SD. Results were obtained in 4-5 different experiments, with three mice per experimental condition. (b) Representative EM image showing the transendothelial transport of 8 nm Au-BSA in control mouse lung. Tracer particles were detected in the vessel lumen, (t), in vesicles open to the luminal side of ECs (v1), in vesicles within the endothelial cytoplasm, (v2), and in vesicles discharging their load in the perivascular space (pvs; v3). Tracer particles did not permeate the IEJ and generated filtration residues (arrowhead), in the luminal introit of the junction. Scale bar: 100 nm. (c) Representative electron micrographs showing transendothelial transport of 8 nm Au-BSA in SH3A-transduced mouse lung specimens as well as nonconventional endocytic/transcytotic intermediates in ECs expressing the SH3A domain of ITSN-1. Open IEJ permeated by 8 nm gold BSA complexes (c1) and heavy association of gold particles with the abluminal exit of an IEJ (c2). Caveolae (c3, c4, arrows) and clathrin-coated pits (c7, arrow) with very long necks, and caveolae with their narrow necks surrounded by staining-dense collars (c5, c6 arrowheads) were frequently observed. A clathrin-coated pit with elongated neck is shown (c8, boxed area, inset). Elongated elements and tubular membranous structures, labeled by gold particles (c1, c9, black boxed structures, c9.1, a9.2) and comprising caveolae and coated pits (c9, c10, white boxed structures) as well as large and uncharacteristic caveolae clusters (c10, arrow and inset). Bars: 200 nm—(c1), (c3), (c7), (c8), (c9.1); 100 nm—(c2), (c4), (c6), (c9), (c8) inset, (c10); 50 nm—(c5), (c9.2).

Once efficient expression of myc-SH3A was achieved, (48 h alter administration of myc-SH3A plasmid/liposome complexes), DNP-BSA was perfused through mouse lung microvasculature and its transendothelial transport was quantified by ELISA, as in [26]. The expression of the SH3A in mouse lung endothelium caused greater than 36% increase in transendothelial transport of DNP-BSA, compared to controls (Figure 2(a)), suggesting disruption of the interendothelial junctions (IEJs). The opening of IEJs was further confirmed by perfusion of 8 nm Au-BSA, at 48 h alter myc-SH3A administration. Electron microscopy analysis of the transport pathway followed by 8 nm Au-BSA from the lumen to the perivascular space in control specimens indicated nearly exclusive association between the tracer and caveolae (Figure 2(b)). After 10 min perfusion, we detected the tracer in the vessel lumen, (Figure 2(b), t), in vesicles open to the luminal side of ECs (Figure 2(b), v1), in vesicles within the endothelial cytoplasm, presumably in transit across the endothelium, (Figure 2(b), v2), in vesicles discharging their load in the perivascular space (Figure 2(b), v3), and in variable number in the perivascular space (not shown). We also determined the location of the tracer in relation to IEJs. We found no tracer in the junctional profiles or associated with their abluminal exit; IEJs were not permeated by the tracer particles that, generated however, filtration residues in their luminal introit, (Figure 2(b), arrowhead). On the contrary, the transendothelial transport of tracer particles in mouse lung endothelium expressing the SH3A revealed open IEJs permeated by Au-BSA complexes (Figures 2(c), and 2(c1), arrows) and in some cases, heavy association of gold particles with the abluminal exit of IEJs (Figures 2(c), and 2(c2)). Interestingly, in SH3A-expressing mouse specimens, caveolae (Figures 2(c), 2(c3), and 2(c4), arrows) and clathrin-coated pits (Figures 2(c), 2(c7), arrow) with long necks, and caveolae whose narrow necks were surrounded by staining-dense collars (Figures 2(c), 2(c5), 2(c6), arrows) were frequently observed. A clathrin-coated pit with elongated neck is shown in Figures 2(c), 2(c8) (boxed area, inset). In addition to caveolae, a large number of elongated elements and tubular membranous structures, many of them labeled by gold particles (Figures 2(c), 2(c1), 2(c9) (black boxed structures), and insets c9.1, c9.2) comprising caveolae and coated pits (Figures 2(c), 2(c9), and 2(c10), white boxed structures) as well as large and uncharacteristic caveolae clusters (c10, arrow and inset c10.1) occupied the intracellular space, in close proximity of the plasma membrane. The diameter of the tubules ranged between 25 nm to 60 nm, while their length varied between 150 nm to 600 nm (*n* = 70 tubules). As previously shown by us [33], the tubular elements may be part of an endosomal tubulovesicular network that may communicate with cell surface via caveolae and clathrin-coated vesicles. The network may be upregulated by expression of the SH3A and acute disturbance of ITSN-1s/Dyn2 interaction. The observation also reflects impaired membrane fission reaction and thus, a stimulatory effect of impaired Dyn2 function on growth of dynamic tubular structures as reported for cells lacking Dyn1/Dyn2 [34]. Delivery of empty liposomes did not affect the morphology of caveolae or ECs (not shown). Based on these findings we concluded that in the presence of SH3A, similar to cultured ECs [8], Dyn2/Dyn2 interactions are stabilized, Dyn2 function is impaired and caveolae cannot detach from the plasma membrane to form free transport vesicles. Thus, impaired caveolae function triggered the expansion of dynamic tubular structures to compensate for deficient transport via caveolae.

**Figure 3:**
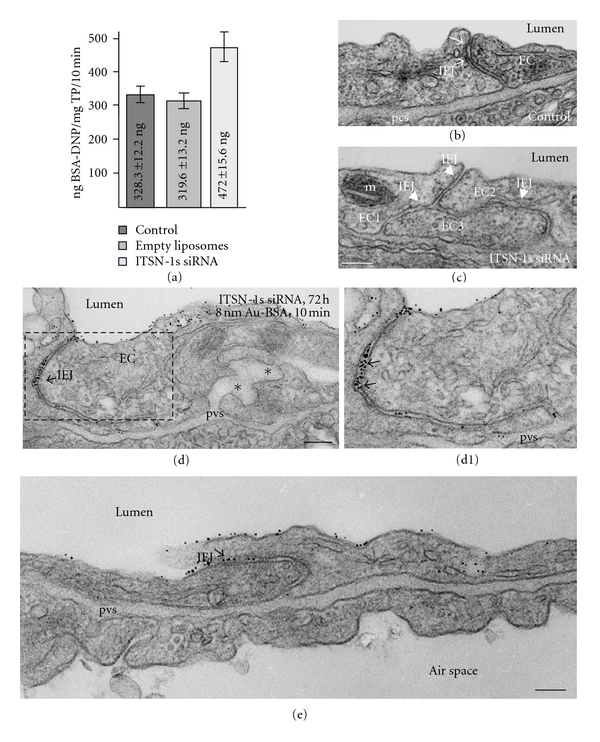
Increased endothelial permeability and impaired interendothelial junctional integrity in mouse lung endothelium acutely depleted of ITSN-1s. (a) ELISA applied on mouse lung lysates of control, empty liposomes, and ITSN-1s siRNA-treated mice, (72 h alter siRNA), perfused with 10 mg/mL BSA-DNP, for 10 min. Results were obtained in 4-5 different experiments, (3 mice/experimental condition), and expressed in ng BSA-DNP/mg total protein/10 min. Bars ± SD. (b, c) EM morphology of IEJs in control (b) and ITSN-1s siRNA (c) mouse lung endothelium. Arrows in B point to the dense protein matrix of the tight junctions sealing the interendothelial space. Arrowheads in (c) point to three open IEJs. EC: endothelial cell; m: mitochondria. Bar: 150 nm. (d, e) Representative electron micrographs showing open IEJs labeled throughout their length by 8 nm Au-BSA particles. Arrows in (d) and magnified d1, point to three-four Au-BSA particles located close to each other in the same plan, indicative of the wide opening of the IEJ. Gold particles are also associated with the abluminal exit of IEJs. Note also the limited number of caveolae and dilation of the pericapillary space (pcs; asterisks). Bars: 200 nm (d); 100 nm (e).

**Figure 4:**
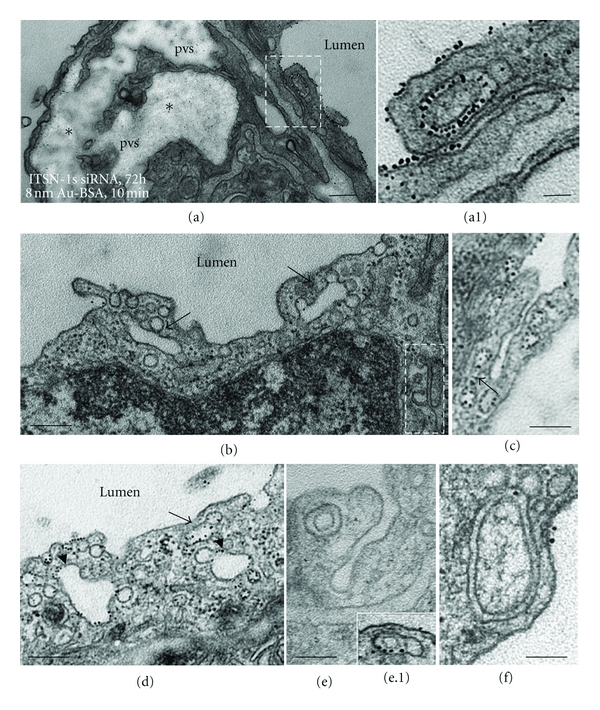
Acute perturbation of ITSN-1s expression induces pleomorphic endocytic/transcytotic intermediates. (a–f) Representative EM images of membranous rings (a, a1, e, e.1, f), tubular elements (b, c, d, arrows) and enlarged endosomes (d, arrowheads), loaded with 8 nm Au-BSA and associated with caveolae-like morphology. Two tubular structures open to the lumen are shown in (c) and (e). The EM image shown in (e) was selected from a mouse lung specimen not perfused with 8 nm Au-BSA. Note also the severe dilation of the perivascular space (pvs) and the proteinaceous edema (a). Bars: 250 nm (a, b); 200 nm (c, d); 150 nm (f); 100 nm (a1, e).

Next, the functional effects of acute ITSN-1s deficiency (72 h) on lung microvascular permeability were assessed by 10 min perfusion of 10 mg/mL DNP-BSA through mouse lung microvasculature and quantifying by ELISA the amounts of DNP-BSA tracer transported to lung interstitia, as above. We observed a significant increase in DNP-BSA transport in the ITSN-1s-deficient mouse relative to controls (i.e., 472 ng DNP-BSA/mg total protein/10 min in knockdown mouse versus 328.3 ng DNP-BSA/mg total protein/10 min, in controls), consistent with endothelial barrier dysfunction, Figure 3(a). We further analyzed by electron microscopy the morphology of IEJs and if the tracer, 8 nm Au-BSA, can escape through open IEJs. Detailed analysis of ultrathin sections prepared from randomly chosen mouse lung specimens acutely depleted of ITSN-1s showed frequently open IEJs. The electron micrograph in Figure 3(c) shows a tricellular junction where one side is obviously open. For the IEJs between the EC2 and EC3, the sectioning of the tissue is less favorable and one cannot conclude on junctional restrictiveness. By contrast, in control specimens (Figure 3(b)) the interendothelial space is sealed by the tight junctions, which are the most apical IEJ structures (Figure 3(b), arrows). These observations strongly demonstrated that the increased permeability for BSA-DNP in the lung vasculature of ITSN-1s-deficient mouse is due, similar to the SH3A expressing mice, to the frequent presence of open IEJs. In ITSN-1s depleted specimens, perfused with 8 nm Au-BSA, the pattern of vesicular labeling was similar to controls, (Figure 2(b)). By distinction to controls, we detected open IEJs heavily labeled throughout their length by Au-BSA particles. Morphometric analysis indicated that about 48% of IEJs counted were open and penetrated by 8 nm Au-BSA complexes (*n* = 227). Under control conditions, mainly in postcapillary venules, we found that approximately 30% of junctions were open to ~6 nm, thus allowing water, ions, and small molecules to take the paracellular pathway to the interstitia, data that are in agreement with [35]. Given the size of Au-BSA complexes (the overall diameter of Au-BSA complexes (gold + BSA molecules) is about 20 nm), we can estimate that the junctions were widely open: if, for example, in some areas, three or four gold particles are found in the same horizontal plan, labeling the junctional space, the opening of the junction could be estimated at about 60 nm–80 nm width. We also detected gold particles associated with the abluminal exit of the junction (Figures 3(d), 3(d1), and 3(e)). Extensive dilation of the perivascular space and rich proteinaceous edema is another phenotypic characteristic of these mouse lungs (Figures 3(d), and 4(a), asterisks). A new interesting observation was the frequent detection of pleomorphic morphological structures/intermediates thought to function in alternative endocytic pathways, such as membranous rings (Figures 4(a), 4(a1), 4(e), 4(e1), 4(f)), many of them heavily loaded with 8 nm Au-BSA particles (Figures 4(a), and 4(a1)), tubules and tubulovesicular elements open to the lumen (Figure 4(b), boxed structures, 4(c), 4(e)) or with no noticeable communication with the extracellular environment (Figures 4(b), 4(c), 4(d), arrows), and enlarged endocytic structures, many of them fused with typical caveolae, apparently discharging their load (Figure 4(d), arrowheads). The rings ranged between 150 nm and 400 nm diameter (*n* = 65 rings) and displayed a narrow lumen, (20 nm ± 4 nm). Finally, significant decrease in caveolae number was also observed in mouse lung ECs deficient of ITSN-1 by reference to controls (Table 1). The findings strongly suggested that inefficient Dyn2 recruitment to the endocytic site caused by ITSN-1s deficiency, and the resultant impaired formation of free caveolae able to function in the transendothelial transport, caused not only disruption of the IEJs, but also prompted the ECs of the mouse lung vasculature to upregulate and employ some alternative/compensatory pathways for endocytic internalization and transendothelial transport (Table 1). Accumulation of tubular elements may also reflect the effect of inefficient Dyn2 recruitment to the endocytic sites, as previously reported for cells lacking Dyn1/Dyn2 [34]. Alltogether, the findings demonstrate that normal ITSN-1s expression is crucial for Dyn-mediated membrane fission reaction of caveolae, for maintaining the caveolae number and for their transport function in lung microvascular ECs. Moreover, experimental manipulation of ITSN-1s expression induces alternative endocytic/transcytotic pathways.

### 3.3. Chronic Inhibition of ITSN-1s Expression Partially Restores Caveolae Number and IEJ Integrity While Maintaining the Activation of Alternative Endocytic/Transcytotic Pathways

**Figure 5:**
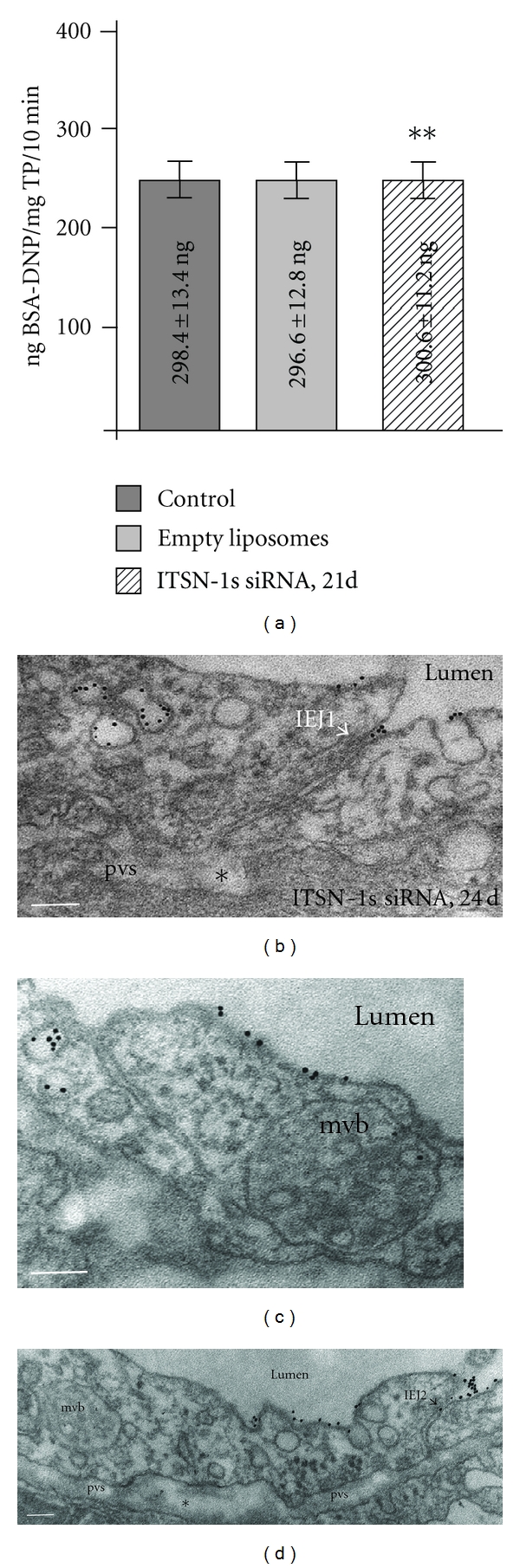
Chronic inhibition of ITSN-1s expression partially restores caveolae number and IEJ integrity. (a) ELISA applied on mouse lung lysates of control, empty liposomes, and ITSN-1s siRNA-treated mice, 24 d alter delivery, perfused with 10 mg/mL BSA-DNP, for 10 min. Results were obtained in 4-5 different experiments, (3 mice/experimental condition), and expressed in ng BSA-DNP/mg total protein/10 min. Bars ± SD. (b, d) Filtration residues in the luminal introit of two IEJs (arrowheads). Mild dilation (*) of the pvs suggests leakiness of the IEJs. Multivesicular bodies (mvb), in close proximity of the plasma membrane, have some of their internal small vesicles, labeled by 8 nm Au-BSA particles (c, d). Bars: 100 nm.

To get more insight into the role of ITSN-1s in regulation of caveolae transport function and endothelial permeability we next used the mouse model with chronic inhibition of ITSN-1s expression for 21 consecutive days (Figure 1(c)). The functional effects of chronic ITSN-1s deficiency on lung microvascular permeability were assessed by 10 min perfusion of DNP-BSA through mouse lung microvasculature and quantifying by ELISA the amounts of DNP-BSA tracer transported to lung interstitia, as described above. The findings were somehow intriguing; transendothelial transport of DNP-BSA as quantified by ELISA, via DNP Ab returns to control levels (Figure 5(a)).

The electron microscopy morphological assessment of interendothelial junctional integrity in mice with chronic inhibition of ITSN-1s expression indicated that the 8 nm Au-BSA particles cannot penetrate the IEJs. Instead, they formed filtration residues in the luminal introit of the junction (Figures 5(b) and 5(d), arrows). Interestingly however, the perivascular spaces showed some dilation and mild edema, suggesting that the IEJs are still leaky, but not permeable to the size of our tracer.

**Figure 6:**
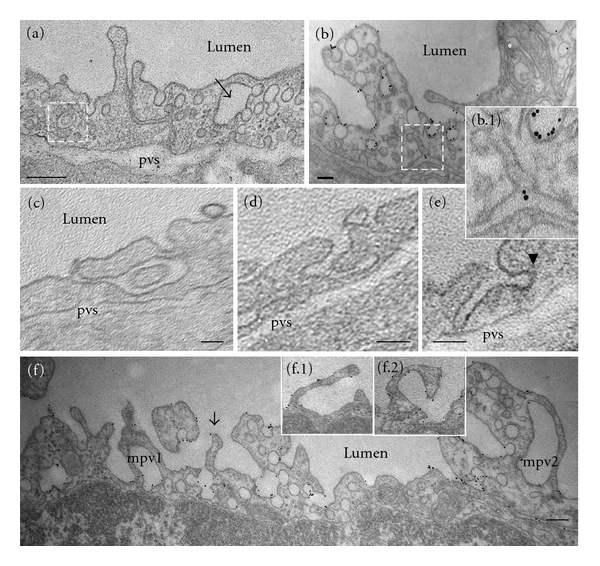
Chronic inhibition of ITSN-1s expression augments the activation of alternative endocytic/transcytotic pathways. (a–e) Representative EM images of membranous rings with diameters between 100 nm to 400 nm, (a, c), free in the cytosol or open to the lumen and tubules (b, d, e), some of them branching or discharging tracer in the perivascular space, (b, boxed structure, and inset), and enlarged endocytic structures, many of them fused with typical caveolae (a). Note the tubular structures are connected to the plasma membrane and displaying at their cytosolic termini caveolae (b1) or coated vesicles (e, arrowhead). Bars: 100 nm (b, c, d); 250 nm (a); 75 nm (e); (f) membrane ruffles (f, arrow, insets f.1, f.2) and large macropinocytic vesicles (mpv1, mpv2) labeled by tracer particles. Bar: 150 nm.

Another interesting observation was the frequent detection of multivesicular bodies (mvb), comprising internal small vesicles of about 50 nm diameter, some of them loaded with 8 nm Au BSA particles (Figures 5(c) and 5(d)). The alternative endocytic pathways and their morphological intermediates are still present and better represented (Figure 6). Membranous rings with diameters between 100 nm to 400 nm, (Figures 6(a) and 6(c)), free in the cytosol or open to the lumen and tubules (Figures 6(b), 6(d), and 6(e)), some of them branching or discharging tracer in the perivascular space, (Figure 6(b), boxed structure, and inset), and enlarged endocytic structures (most probably secondary lysosomes), many of them fused with typical caveolae (Figure 6(a), arrow) were frequently detected. Often, the tubular structures connected to the plasma membrane displayed at their cytosolic termini caveolae (Figures 6, 6(b1)) or coated vesicles (Figure 6(e), arrowhead). We have also found membrane ruffles (Figure 6(f), arrow, and insets (f.1), (f.2)) and macropinocytic vesicles (Figure 6(f), mpv1, mpv2), involved in tracer uptake, as shown by their gold labeling, and thus, possibly compensating for deficient caveolae transport.

Morphometric analyses indicated that the number of endocytic/transcytotic structures is slightly increased at day 24, compared to 72 h time point, with the membranous ring/tubules labeled by Au-BSA showing a further increase to 14-fold, compared to controls (Table 1). Caveolae number is partially restored, only 17.8% decrease, by reference to control conditions. Collectively, these observations emphasize that physiological levels of ITSN-1s are critical for transendothelial transport via caveolae and paracellular permeability *in vivo*.

## 4. Discussion

We have shown that ITSN-1s is required for efficient transendothelial transport via caveolae and provided *in vivo* ultrastructural evidence to suggest that modulation of ITSN-1s expression activates the paracellular pathway as well as a previously described, but less understood endocytic tubulovesicular network and membranous rings that seem to efficiently compensate for deficient caveolae transport function. So far, limited evidence regarding nonconventional endocytic mechanisms were partly obtained in cultured cells mainly by studies of toxins and viruses [36–40]. In this study, using a murine model expressing the myc-SH3A domain of ITSN-1s, capable of regulating Dyn2 activity at the endocytic sites [8], and mice acutely (72 h) and chronically (21 d) depleted of ITSN-1s via siRNA, we directly demonstrate that by interfering with expression of ITSN-1s, a key partner of Dyn in endocytic membrane fission, the caveolae function was disrupted and alternative endocytic/transcytotic pathways and their morphological intermediates were activated. Prior studies by others and us indicated that ITSN-1s functions by recruiting Dyn and regulating its function at the endocytic site [6, 8, 16, 21–23, 41]. Studies in cultured ECs, complemented by *in vitro* assays demonstrated that the SH3A domain of ITSN-1s stimulates both basal and assembly-stimulated GTPase activity of Dyn2, as well as Dyn2 ability to form oligomeric structures [8]. In the presence of SH3A at the endocytic site, Dyn2/Dyn2 interactions are stabilized, the lifetime of assembled Dyn2 is prolonged, and despite continuous GTP hydrolysis, Dyn2 oligomers cannot disassemble. Membrane fission reaction and the subsequent detachment of caveolae from the plasma membrane leading to the formation of free vesicular carriers were inhibited. Here, we extended these studies and expressed the SH3A of ITSN-1s in mouse lung endothelium, to address the *in vivo* regulatory role of ITSN-1s on membrane fission and Dyn itself. Similar to cultured cells, we see impaired caveolae release from the plasma membrane, caveolae necks surrounded by staining-dense collars as well as elongated elements and tubular membranous structures, associated with caveolae-like morphology. In addition, the deficient transcellular transport via caveolae caused opening of the IEJs and activation of the paracellular pathway, consistent with a functional crosstalk between the trans- and paracellular transport pathways in mouse lung endothelium. These observations support the *in vivo* role of ITSN-1s, via its SH3A domain, in regulation of Dyn2 function at the neck of caveolae and the importance of ITSN-1s/Dyn2 interaction in caveolae internalization and trafficking.

Acute siRNA-mediated knockdown of ITSN-1s, 72 h alter liposomes/siRNA delivery caused 59% decrease in caveolae number, disruption of IEJs, leading to 43% increase in DNP-BSA transendothelial transport, compared to controls. Without ITSN-1s, Dyn2 a major interacting partner of ITSN-1s and essential player in detachment of caveolae from the plasma membrane is not efficiently recruited to the endocytic site and thereby, caveolae detachment from the plasma membrane and formation of free vesicular carriers is disrupted [19, 23]. Similar to Dyn, other endocytic proteins may be mislocated and may interact less efficiently affecting the dynamic nature of endocytic complexes [23]. Thus, regardless of the molecular mechanisms involved—impaired Dyn function, in the case of the SH3A expression, or inefficient Dyn recruitment to the endocytic site, in the case of ITSN-1s knockdown—the membrane fission reaction is inhibited and caveolae detachment from the plasma membrane does not occur. ITSN-1s with its five SH3 domains is critical in maintaining high Dyn concentration and thus, regulation of its GTPase activity and oligomerization at the endocytic site.

Transendothelial transport (transcytosis) via caveolae is the dominant mechanism of transendothelial transport; the number of clathrin-coated vesicles in ECs is relatively small and their contribution to the internalization process is minor [6, 42, 43]. In ECs the true caveolae (vesicular carriers Cav1 positive) represent ~95% of the whole vesicular population and the non-Cav1 and nonclathrin vesicles identified in the Cav1 null mouse are poorly represented and not yet well defined as a Cav1 and clathrin-independent vesicular transport system [44, 45]. Our studies confirmed that indeed caveolae are the main vesicular carriers of ECs functioning in transendothelial transport. Similar to Cav1 null mice, that lack morphologically identifiable caveolae, altered expression of ITSN-1s, by decreasing caveolae number and impairing their transport function, activated compensatory transport pathways. We showed here that increased paracellular transport of tracer particles with diameter between 6 nm (DNP-BSA) to 20 nm (Au-BSA) occurs in the ECs of mice with acute modulation of ITSN-1s levels, while the high frequency of pleomorphic tubulovesicular and ringlike structures labeled by Au-BSA, implied their direct participation in tracer uptake and cellular trafficking. ITSN-1s deficiency was able not only to induce activation of classical interendothelial pathway, but also to modulate the opening of the junctions. Under conditions of ITSN-1s chronic inhibition, the paracellular pathway is still leaky but not permeable to 18 nm diameter tracer. Apparently, ECs deficient of ITSN-1s limit the activation of the paracellular pathway to small molecules, while the transport of macromolecules (Au-BSA and DNP-BSA) reach similar values as the controls. This apparent return of junctional integrity may be part of a general protective mechanism against inflammation and lung injury. Moreover, the increased presence of pleomorphic tubulovesicular and ringlike structures labeled by Au-BSA suggests their augmented participation in tracer uptake and cellular trafficking. To our knowledge, this is the first report showing the *in vivo* modulation of alternative transport pathways.

A large intracellular tubulovesicular network of endosomal origin that extended all the way from the nucleus to the plasma membrane and involved in receptor recycling has been previously reported [46]. Later on, a similar system was characterized by Palade's group and shown to comprise G-protein-coupled receptors for thrombin and platelet activating factor [33, 40, 47, 48]. Structurally, it was shown that this tubulovesicular network does not colocalize with the early endosomes, Golgi and endoplasmic reticulum. Moreover, the network was shown to communicate with both extracellular environment and nuclear compartment of ECs. HRP uptake studies indicated that HRP labeled this tubular network, in just minutes of treatment, consistent with the idea it participates very early in the internalization process [33]. In the mouse models used in this study, the alternative intracellular transport routes are upregulated; deficient recruitment and impaired Dyn function at the endocytic sites due to SH3A expression or ITSN-1s depletion may favor the formation of tubular structures as shown in cells with conditional Dyn1/Dyn2 knockout [34]. In addition, an impaired coordination between membrane invagination and vesicle detachment may lead to formation of large invaginations. Both ITSN-1s and 2 directly interact with the FCHo1/2 Bar domain containing proteins [24, 49]. While no direct functional link has been established between ITSN-1s and F-BAR domain containing proteins in caveolae budding, this interaction is likely to account for the altered caveolae morphology within the tubulovesicular structures and caveolae clusters. Interestingly, chronic inhibition of ITSN-1s shows no increase in the DNP-BSA transported to the interstitia. Widening of IEJs is limited. The number of nonconventional endocytic structures was greater, with the membranous ring/tubules showing a 2-fold increase, compared to 72 h ITSN-1s knockdown.

While our results can be explained by a model where ITSN-1s modified expression and Dyn2 impaired function may favor membrane tubulation, a direct action of ITSN-1s on actin polymerization cannot be ruled out. We have shown that the temporal and spatial actin polymerization and remodeling subadjacent to the plasma membrane plays an important role in caveolae internalization [50]. ITSN-1s binds via its SH3A the N-WASp, [51], an actin-nucleating factor that acts at the plasma membrane upstream of Arp2/3 complex [52]. The physiological significance of WASp interaction with short ITSN-1, which does not possess the DH-PH tandem as the long ITSN isoforms, is still not understood. Impaired caveolae internalization and trafficking may also have effects on cellular signaling. The upregulation of endocytic membranous structures may provide distinct signaling platforms [53], capable of partially compensating for caveolae signaling. Moreover, the caveolae number was partially restored, suggesting that the highly homologous ITSN-2s isoform may partially compensate for ITSN-1s chronic deficiency, a question that remains to be addressed in future work.

Caveolae and Cav1are involved in important cellular functions such as proliferation, apoptosis, cell differentiation, cell migration, endocytosis, and transcytosis [44, 54, 55]. Caveolae are present in all cell types in the lung, including ECs, epithelial cells, smooth muscle cells, fibroblasts, telocytes, and macrophages [55, 56]. Recent research implicated caveolae and Cav1 in pathogenesis of human lung disease [45, 57, 58], neurodegenerative diseases [59], and lung cancer [60, 61]. Not only Cav1, but also the ubiquitously expressed ITSN-1s, a major protein of lung tissue, has been related to lung inflammatory conditions [62], to endosomal disorders associated with the earliest pathogenesis of Alzheimer's disease and Parkinson's diseases [63] as well as cancer [64, 65]. Thus, a better understanding of the endocytic function of ITSN-1s and how it regulates caveolae internalization and trafficking may provide new insights for understanding of human disease and give new opportunities for novel targets in therapeutic approaches.

In conclusion, our results provide new information on the *in vivo* role of ITSN-1s as a scaffold, recruiter and regulator of caveolae endocytic machinery and strengthen the evidence for a functional endocytic tubulovesicular network induced by ITSN-1s deficiency.

## Figures and Tables

**Table 1:** Chronic inhibition of intersectin-1s expression causes the activation of alternative endocytic/transcytotic pathways and partially restores caveolae number.

Endocytic/transcytotic structures	Control	ITSN-1s siRNA, 72 h	ITSN-1s siRNA, 24 d
Caveolae open to the lumen*	106.6 ± 9.5	50.46 ± 4.8	87.15 ± 5.9
Caveolae apparently free in the cytosol	173.5 ± 12.0	77.41 ± 6.3	143.0 ± 9.5
Total caveolae (luminal and free in the cytosol)	280.1 ± 21.5	127.86 ± 11.1	230.14 ± 15.4
Abnormal endocytic structures (enlarged endosomes)	2.65 ± .34	11.29 ± 2.7	14.93 ± 3.8
Caveolae clusters	3.95 ± .4	5.64 ± 1.6	11.3 ± 2.5
Membranous rings	1.33 ± .04	9.47 ± 2.4	12.8 ± 2.8
Tubules	.88 ± .05	4.0 ± 1.3	18.84 ± 3.4

*Results are normalized per 100 *μ*m EC length.

## Abbreviations

Ab: Antibody
Au-BSA: Gold-bovine serum albumin
caveolin-1: Cav1
DNP-BSA: Dinitrophenylated-bovine serum albumin
Dyn: Dynamin
EC: Endothelial cell
IEJs: Interendothelial junctions
ITSN-1s: Intersectin-1s.
